# Freudian Theory and Consciousness: A Conceptual Analysis**

**DOI:** 10.4103/0973-1229.77437

**Published:** 2011

**Authors:** Avinash De Sousa

**Affiliations:** * *Consultant Psychiatrist and Psychotherapist, Mumbai, India.*

**Keywords:** *Consciousness*, *Ego psychology*, *Freud*, *Psychoanalysis*

## Abstract

This paper aims at taking a fresh look at Freudian psychoanalytical theory from a modern perspective. Freudian psychology is a science based on the unconscious (id) and the conscious (ego). Various aspects of Freudian thinking are examined from a modern perspective and the relevance of the psychoanalytical theory of consciousness is projected. Do psychoanalysis and the unconsciousness have something to teach us about consciousness? Approaching Freud from a historical, psychoanalytical, anthropological and sociological perspective, we need to look at how Freudian theory may contribute to a better understanding of consciousness. We also need to look at psychoanalytical psychotherapy and its contribution to a better understanding of body-mind dualism and consciousness as a whole. Ego psychology is considered in the present day context and it is synthesized with various psychological studies to give us a better understanding of consciousness.

## Introduction

“If often he was wrong and, at times absurd, to us he is no more a person now but a whole climate of opinion under whom we conduct our different lives…”

(*W.H. Auden, In Memory of Sigmund Freud) (Auden and Mendelson, 1991*)

Despite distorted understandings of Freudian views and despite periodic waves of Freud bashing, Auden’s assessment remains essentially correct. Freud’s influence continues to be enormous and pervasive. He gave us a new and powerful way to think about and investigate human thought, action and interaction. He often made sense of the ranges that were neglected or misunderstood. Although one might wish to reject or argue with some Freudian interpretations and theories, his writings and insights are too compelling to simply turn away. There is still much to be learned from Freud (Neu, 1991). Much to be learned in relation to issues in contemporary philosophy of mind, moral and social theory. The special characteristics of unconscious states including their relations to states described by modern psychology and the relevance of the Freudian unconsciousness to questions concerning the divided or multiple self is equally important. This paper looks at the connection between Freudian theory/concepts and modern day conceptualisation of consciousness.

## Is the Freudian unconscious relevant in the light of modern day consciousness?

Psychoanalysis regarded everything mental being in the first place unconscious, and thus for them, consciousness might be present or absent. This of course provoked a denial from philosophers for whom consciousness and mental were identical and they could never conceive of an absurdity such as an unconscious mental state. Reasons for believing in the existence of the unconsciousness are of course empirical, but the question as to what most fundamentally distinguishes the Freudian unconscious is a conceptual one. It is very important that one understands the nature of the unconsciousness in broad holistic terms rather than the fine details that Freud gave, and also one must follow the coherence of such a concept to understand our present day understanding of consciousness (Freud, 1912; Ricoeur, 1970).

The qualified specialization of consciousness that can be located in ordinary thought about the mind provides a source of motivation that is free from conceptual confusion. The analysis of what it is to be in consciousness has a further importance for the concept of unconscious mentality. If one assumes that all mental states are conscious alone, we will take a highly sceptical stand on Freudian theory and the topographical model of the mind proposed by him (Laplanche and Pontalis, 1983). For example, mental states like beliefs and values do not exist solely by virtue of the consciousness in them. Freud’s notion of unconscious mentality is arrived at by pressing the distinction of mental states from consciousness and combining it with the topographical model where all the psychological locales are spoken of as existing independently from their members at any given moment (Freud, 1915; Freud, 1937).

In William James’s *The Principles of Psychology* (James, 1890), the concept of unconscious mentality is considered in terms of its role as a necessary concomitant of what James calls the mind stuff theories by which he means theories that regard mental states as empirically analysable compounds.

It would now be helpful to spell out more precisely various conceptions of the psychoanalytic concept of the unconsciousness in terms of successive degrees of independence from the concept of consciousness.

Unconsciousness may be entirely composed of ideas that were previously conscious and have been repressed. This would meet the Lockenian condition on mentality, that is, there can be nothing in the mind that has not been previously in awareness (Ricoeur, 1970).

Unconsciousness may be perceived as entirely composed of, or at least as including some ideas that were not originally conscious but that could become conscious (Sears, 1943).

The last of these conceptions matches the unconsciousness as described in the writings of Melanie Klein and Wilfred Bion (Bion, 1984; Dryden, 2004), but it is also most probably attributable to Freud. The evidence for the same comes from Freud’s explicit statements that the concept of unconsciousness is broader than that of the repressed and also is made up of a phylogenetic heritage and primal fantasies (Freud, 1938).

A different question now needs to be addressed. It has been supposed that positive reason to believe in the existence of unconsciousness may come, and does in fact come from the notion that unconsciousness is necessary as data of consciousness have very large number of gaps in them (Freud, 1915). Consciousness is characterized by a special kind of unity, on account of which it does not tolerate gaps of any kind. We could interpret Freud’s notion in terms of gaps in self-explanation. These gaps are as such fully psychological in nature and they occur at points where we would ordinarily expect an intentional psychological explanation to be available and in this way, they stand apart from other merely nominal gaps in ordinary psychological explanation (for example, the impossibility of explaining how it is that one ordinarily remembers something).

Freud in his topographical model never looked at the mind to be built up of a number of agencies or systems, but rather these were terms used in a very special way, and it is a further puzzle as to what precisely Freud wanted them to signify (Freud, 1923). Consciousness and unconsciousness are not inimical properties and they are not intrinsically antagonistic to each other. Conflict between them is not regarding their status but because of the particular character of the contents of unconsciousness and their consequent connection with repression (Wollheim, 1973).

Many questions remain unanswered, but it is fitting to conclude that consciousness and unconsciousness are both a set of states with representational content distinguished by special features which need not be regarded as propositional attitudes, characteristically endowed with phenomenology but attributed in a spirit of pure plain psychological realism (Archard, 1984).

## Relationships Between Freudian Theory and Cognitive Psychology with Reference to Consciousness

Though over a century has elapsed since Freud first proposed his theory, there has been very little comparison between Freudian theory and its links to nonpsychoanalytic academic psychology. The choice of cognitive psychology in this discussion stems from the fact that cognitive theory and cognitive psychology have a basis in almost all facets of modern psychology. Though cognitive psychology has explained many areas unknown to us 50 years earlier, one must admit that no other theorist ever constructed a conceptual and metatheoretical framework like Freud did, in order to understand psychological questions. No theory so far has ever provided a theory conceptually superior to Freud’s (Reiser, 1984).

Freud reduced the role of consciousness to that of an epistemological tool to know about certain areas of one’s mental state, removing all ontological implications. The evidence available in his time suggested that some mental states might exist outside ones awareness. Thus, Freud had to reject the principle that all mental states are conscious (ontological), but he retained the principle that all conscious states are accessible to awareness (epistemological). The demotion of consciousness to a purely epistemological role leads to serious failure, both by Freud and other theorists. In the transformation of psychology from a science of consciousness to a science of mental representations, there has been a gain in theoretical power, but there has been a loss of something of great value. Psychologists may in fact be avoiding the problem that made the mental realm so puzzling in the first place, the problem of consciousness, and thereby ignoring the mystery that is at the heart of the nature of meaning and mind (Grunbaum, 1984; Holt, 1989; Roth, 1998).

The term ‘conscious’ refers to an irreducible and irreplaceable phenomenon, no matter what the name. Terms such as awareness, reflective awareness, phenomenal awareness and phenomenal representation have all been used to refer to the same thing. Awareness has been used to refer to what we mean when we are at the moment conscious of something but also refers to the latent knowledge of something. The term conscious, unless burdened with additional meaning, may serve to mean what is immediately, subjectively and introspectively given in experience. We may be thus conscious of a rational abstract idea, an obsessional preoccupation or even a hallucination. We are conscious in psychosis, dissociative states, in intoxication and so forth. But each of these represents a quite different mental organisation of experiences, obeying different principles of organisation and existing on different levels of categorization and abstraction (Kihlstrom, 1987).

We shall now take a look at the confusion, both terminological and conceptual, that dogged Freudian thought as well as contemporary cognitive psychology. Freud always struggled with what has been called an adjectival and substantive use of the term conscious. It simply means that the term conscious idea denotes an idea that is directly, subjectively given and capable of being introspected, although it need not be. The experience can be conscious in a variety of different states, i.e., waking alert state, dream state, psychotic state and so on. It is better to refer to the above states as psychological states rather than different states of consciousness. The experience of consciousness may be different in each state but consciousness as a subjective, introspective given, is indivisible no matter what the state of consciousness. But the principles of organisation, levels of categorization and abstraction affecting or producing the experience may be different.

Cognitive psychology has not been immune to confusing and ambiguous uses of the term conscious and consciousness. If consciousness can occur in a variety of psychological states regardless of the principles of organisation, what purpose does being conscious serve and what shall then be the special conditions needed for consciousness to occur? What is the role that consciousness must play in our lives, apart from the operation of the different principles of organisation and levels of abstraction?

Freud gave consciousness the quality and capacity to transform experienced activity into unconscious states, similar to how different forms of energy are interchanged in physics. It could also play a part in inhibiting and restricting certain thoughts from becoming conscious. It also served the purpose of transforming quantities of unconscious excitation into qualitative experiences of pleasure and unpleasure (Freud, 1900; Hartmann, 1964).

## Conclusions [see also Figure 1]

**Figure:**
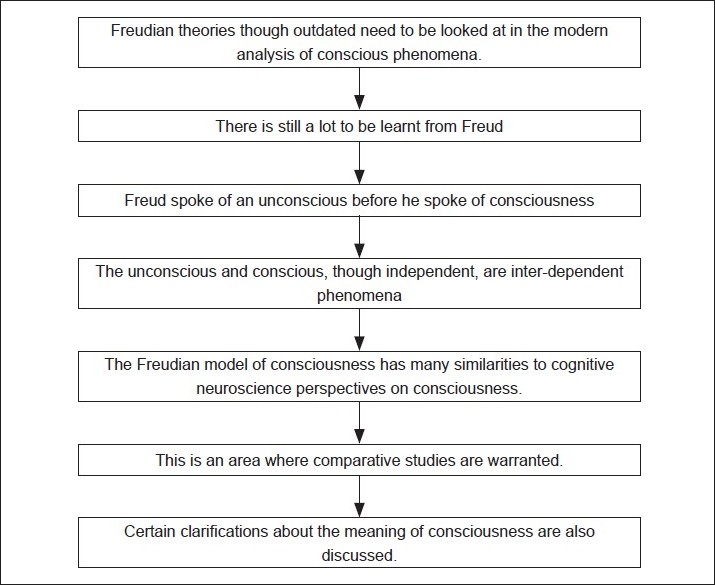
Flowchart of the paper

Whether psychoanalytic and cognitive science views of the consciousness are fraternal or identical twins, we do not know, but they were certainly reared apart from one another. The psychoanalytic twin was raised in the consulting room, exposed to primal scenes, intrapsychic conflict and the risky improvisations of clinical work, whereas the cognitive twin was raised in the scientific laboratory where calm and order prevailed. There is no doubt that the cognitive and psychoanalytic views are different and come out of different traditions (Shervin and Dickman, 1980). Cognitive science focusses on motive, affect and conflict, whereas psychoanalysis focusses on conflict and underlying psychological processes. There are in fact convergences between these two radically different views but from a holistic perspective. They follow a similarity in the nature of the problems they address, though at first look they seem to be far apart.

The newer developments in the field of cognitive science dealing with levels of categorisation and organisation will be of immense value in studying the hierarchical relationship between unconscious and conscious experiences. The chasm between the consulting room and scientific laboratory may soon narrow. We are now at a stage where we must broaden and deepen the scientific investigation of consciousness and conscious states in a way never done before. We need to apply our imagination and good will while being open minded and flexible at the same time.

### Take home message

Freudian theory needs to be given a fresh look. Though considered outdated by some, it has a lot to offer to modern theories of consciousness. Insights from Freudian theory are relevant to modern day concepts of consciousness in cognitive neuroscience. Consciousness and unconsciousness are both independent and interdependent phenomena and their study will yield a different perspective on the evolution of conscious phenomena.

## Questions That This Paper Raises

1. Is Freudian relevant today, or must it be done away with?
2. Does Freudian theory play a role in explaining our modern day concept of consciousness?
3. Does the Freudian concept of ‘conscious’ relate at least conceptually to the concept of ‘conscious’ in cognitive psychology?
4. Do parts of Freudian theory have resemblance with modern day cognitive psychology and its theories?
5. Should qualitative research on Freudian concepts be carried out in the light of modern theories of consciousness?
6. Should modern methods of neuroimaging and neuroscience in the light of new data be used to validate Freudian models of conscious phenomena?

## About the Author

*Avinash De Sousa is a consultant psychiatrist and psychotherapist with a private practice in Mumbai. He is an avid reader and has over 40 publications in national and international journals. His main areas of interest are alcohol dependence, child and adolescent psychiatry, mental retardation, autism and developmental disabilities. He is also the academic director of the Institute of Psychotherapy Training and Management, Mumbai. He teaches psychiatry, child psychology and psychotherapy at over 18 institutions as a visiting faculty.*
